# Disease-causing point-mutations in metal-binding domains of Wilson disease protein decrease stability and increase structural dynamics

**DOI:** 10.1007/s10534-016-9976-7

**Published:** 2016-10-15

**Authors:** Ranjeet Kumar, Candan Ariöz, Yaozong Li, Niklas Bosaeus, Sandra Rocha, Pernilla Wittung-Stafshede

**Affiliations:** 1 Department of Biology and Biological Engineering, Chalmers University of Technology, 41296 Gothenburg, Sweden; 2 Department of Chemistry, Umeå University, 90187 Umeå, Sweden

**Keywords:** Wilson disease, ATP7B, Metal-binding domain, Thermal stability, Circular dichroism, Molecular dynamics

## Abstract

After cellular uptake, Copper (Cu) ions are transferred from the chaperone Atox1 to the Wilson disease protein (ATP7B) for incorporation into Cu-dependent enzymes in the secretory pathway. Human ATP7B is a large multi-domain membrane-spanning protein which, in contrast to homologues in other organisms, has six similar cytoplasmic metal-binding domains (MBDs). The reason for multiple MBDs is proposed to be indirect modulation of enzymatic activity and it is thus intriguing that point mutations in MBDs can promote Wilson disease. We here investigated, in vitro and in silico, the biophysical consequences of clinically-observed Wilson disease mutations, G85V in MBD1 and G591D in MBD6, incorporated in domain 4. Because G85 and G591 correspond to a conserved Gly found in all MBDs, we introduced the mutations in the well-characterized MBD4. We found the mutations to dramatically reduce the MBD4 thermal stability, shifting the midpoint temperature of unfolding by more than 20 °C. In contrast to wild type MBD4 and MBD4D, MBD4V adopted a misfolded structure with a large β-sheet content at high temperatures. Molecular dynamic simulations demonstrated that the mutations increased backbone fluctuations that extended throughout the domain. Our findings imply that reduced stability and enhanced dynamics of MBD1 or MBD6 is the origin of ATP7B dysfunction in Wilson disease patients with the G85V or G591D mutation.

**Electronic supplementary material:**

The online version of this article (doi:10.1007/s10534-016-9976-7) contains supplementary material, which is available to authorized users.

## Introduction

Copper (Cu) is found in the active sites of many essential proteins that participate in key cellular reactions (Huffman and O’Halloran 2001; Puig and Thiele 2002; Harris 2003). However, free Cu ions are potentially toxic for cells since they are capable of producing reactive oxygen species which results in oxidative deterioration of biological molecules (Valko et al. 2005). To avoid Cu toxicity, the intracellular concentration of Cu is regulated via dedicated proteins that facilitate uptake, efflux as well as distribution of Cu to Cu-dependent proteins and enzymes (Festa and Thiele 2011; O’Halloran and Culotta 2000; Robinson and Winge 2010). In the human cytoplasm, after the uptake of Cu ions (Ohrvik and Thiele 2014), the Cu chaperone Atox1 transports the metal to the membrane-bound ATP7A and ATP7B (Menke’s and Wilson disease proteins, respectively), two homologous P_1B_-type ATPases located in the trans-Golgi network. Once transferred to ATP7A/B, the Cu ion is channeled to the lumen of the Golgi where it is loaded onto specific Cu-dependent proteins and enzymes.

ATP7A/B are multi-domain membrane proteins with six cytoplasmic metal-binding domains (MBDs) connected by peptide linkers of various lengths constituting the N-terminal tail (Lutsenko et al. 2007). Each MBD in ATP7A/B, as well as Atox1, has a ferredoxin-like α/β fold and a surface-exposed invariant CXXC motif (X = any residue) in which a single Cu can bind to the cysteine sulfurs. In contrast to humans, bacterial and yeast P_1B_-type ATPases have only one or two MBDs. The reason for the presence of multiple MBDs in ATP7A/B has been proposed to be connected with the regulation of Cu transfer to the Golgi lumen and Cu-mediated protein trafficking between the Golgi and the plasma membrane (Forbes et al. 1999; LeShane et al. 2010; Hasan et al. 2012; Huang et al. 2014). During the catalytic cycle which requires ATP hydrolysis and transient phosphorylation, ATP7A/B are likely to undergo significant conformational changes and changes in domain–domain interactions (Lutsenko et al. 2007). Since there is no high-resolution structural information on the arrangement of the six MBDs within full length ATP7A/B, it is unclear how these domains are arranged relative to each other at different stages of the catalytic cycle. Because Atox1 can deliver Cu to the MBDs (Pufahl et al. 1997; Wernimont et al. 2000; Achila et al. 2006; Banci 2006, 2008, 2009a, 2009b), one may speculate that Cu-triggered conformational changes among these domains might initiate the catalytic cycle upon Atox1-mediated Cu delivery (Mondol et al. 2016). Nonetheless, it remains unclear if the direct path for Cu goes through the MBDs or, if Atox1, like its bacterial homolog (Gonzalez-Guerrero and Arguello 2008), delivers Cu directly to a binding site at the membrane-spanning parts of ATP7A/B. It has been shown in vitro that several MBDs can be deleted/mutated without loss of Cu transport activity, but the presence of at least one MBD appears to be required (Forbes et al. 1999; Morin et al. 2009).

**Fig. 1:**
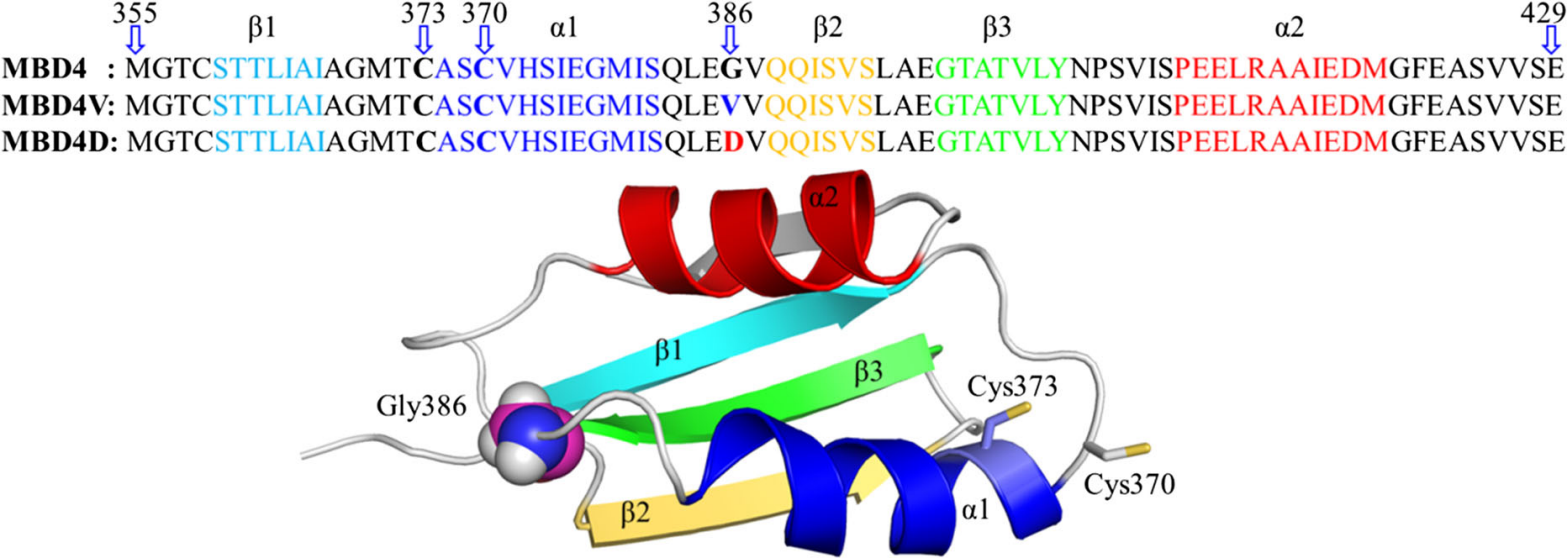
Amino acid sequence and 3D structure of MBD4 variants. The color scheme of the primary sequences follows that of 3D structure; secondary structures are differently colored. Gly386 is presented as a sphere and the Cu-binding Cys are shown in stick representation

Mutations in ATP7B constitute the basis of Wilson disease, a genetic disorder where Cu accumulates in tissues, often resulting in neurological or psychiatric symptoms together with liver dysfunction (Thomas et al. 1995; Gitlin 2003). Over 300 missense mutations in ATP7B have been described in different patients (Huster et al. 2012), with the most common mutation being H1069Q (Caca et al. 2001; Rodriguez-Granillo et al. 2008). Taken into account the proposed indirect role of the MBDs for ATP7B function, it is noteworthy that at least 3 disease-causing missense mutations have been located in the MBDs: G85V in MBD1; L492S in MBD5; and G591D in MBD6 (Hamza et al. 1999; Hsi et al. 2008) with all three residues being strictly conserved. Using a functional assay in yeast, the introduction of G85V or L492S mutations was found to result in an inactive ATP7B (Huster 2012). Therefore, it has been suggested that these MBD mutations will cause domain instability (Hamza et al. 1999), but this has not been tested experimentally. L492 is situated in the core of the fifth metal binding domain and mutation at this position can clearly have dramatic structural effects. In contrast, G85 and G591 correspond to a conserved Gly found in all six MBDs (Hamza et al. 1999) situated in a surface-exposed turn opposite to the Cu binding site (Fig. 1). The predicted effects of mutations at this position are still unclear as one may argue that a loop residue would not be particularly important for the overall domain stability.

To investigate the biophysical impact of mutations of the conserved Gly in the MBDs, we here mutated the corresponding Gly (G386) in MBD4 (which we have characterized extensively previously (Niemiec et al. 2012, 2014, 2015)) to Val (V) and Asp (D) and compared the resulting variants to the wild-type protein in vitro and in silico. We discovered that both variants have dramatically lower thermal stability in parallel with increased structural dynamics as compared to the wild-type MBD4.

## Materials and methods

### Protein preparation

MBD4 wild-type construct in a pET21b vector (Niemiec et al. 2012, 2015) was transformed into BL21 (DE3) plysS (Novagen) cells. Transformants were first grown to an OD_600_ of 0.6, and then induced with 1 mM isopropyl β-D-1-thiogalactopyranoside (IPTG) and grown overnight at 25 °C. The cells were lysed using sonication in 20 mM Tris–HCl buffer pH 8.0 with 2 mM 1,4-Dithiothreitol (DTT) and in the presence of protease inhibitor cocktail (Roche). The centrifuged lysate was loaded onto 5 mL HiTrap Q HP anion exchange column (GE Healthcare) and was eluted by linear gradient with 1 M NaCl in 20 mM Tris–HCl buffer pH 8.0 with 2 mM DTT. The fractions containing MBD4 were combined and concentrated with Ultra-15 Ultracel 3 K centrifugal filter devices. The concentrate was loaded onto Hiload 16/600 Superdex 75 column (GE Healthcare) and retrieved in 10 mM Tris–HCl buffer pH 8.0 and 1 mM DTT. For MBD4D (MBD4 with G386D mutation, corresponding to G591D in MBD6) and MBD4V (MBD4 with G386V mutation, corresponding to G85V in MBD1), tagged versions (several tags including a repressor protein and a His-stretch with a Caspase 7 cleavage site) in pET3a constructs were transformed into BL21 (DE3) plysS (Novagen) cells, which were then grown in similar culturing conditions as used for the wild-type MBD4. The cells were harvested by centrifugation, re-suspended in Buffer A (20 mM Tris–HCl pH 8, 50 mM NaCl and 2 mM DTT) and stored at −80 °C. For purification, the cell suspension was thawed and a protease inhibitor cocktail was added. After incubation on ice for 30 min, the cells were sonicated and centrifuged. The supernatant was filtered through a 0.2 µm filter and applied to 5 mL Hi Trap Ni–NTA column (GE healthcare). After washing the column with 5 column volume of buffer A containing 40 mM imidazole, the protein was eluted with buffer A having 250 mM imidazole. The tag was cleaved by adding Caspase 7 at the ratio of 1:100 (caspase 7: MBD4 mutant w/w) in the presence of 20 mM β-Mercaptoethanol and incubated overnight at 4 °C. The cleaved protein was then loaded onto a 5 mL HiTrap Q HP anion exchange column (GE Healthcare) and was eluted by linear gradient with buffer A containing 1 M NaCl. The eluted fractions were pooled, concentrated and loaded onto a Hiload 16/600 superdex 75 column (GE Healthcare). The obtained pure protein was finally stored at −80 °C in 20 mM Tris–HCl buffer pH 8.0 with 50 mM NaCl and 1 mM DTT. For all purified proteins, the sample purity was confirmed by a single band on a SDS-PAGE gel (Fig. S1) and a single elution peak in size exclusion chromatography. The concentration of the proteins was determined using ε_280_ = 1490 M^−1^ cm^−1^ (MBD4 has one Tyr).

### Circular dichroism spectroscopy

Far-UV circular dichroism (CD) spectra of MBD4 wild type and variants (65 µM) in 20 mM Tris–HCl buffer pH 8 and 50 mM NaCl were recorded using a Chirascan CD spectrometer (Applied Photophysics) in 1 mm quartz cuvette at 6 °C, with 1 nm steps, a bandwidth of 1 nm and a time-per-point of 1 s. The spectra were averaged 5 times and baseline subtracted. The thermal unfolding of the proteins (65 µM) was monitored at 222 nm in stepped ramp mode from 6 to 98 °C (with a temperature-sensitive probe inserted into the cuvette) with a bandwidth of 1 nm, 17 s per data point, and three repeats for each set. Experiments were performed in the absence and presence of equimolar amounts of copper to WD4. A fivefold molar excess of DTT to protein was maintained in all experiments in order to facilitate copper reduction prior to protein loading. The reversibility of protein thermal denaturation was tested by CD-monitored cooling experiments and incubation at fixed temperatures. The CD data sets are reported as mean residue molar ellipticity (degrees M^−1^ m^−1^).

### Molecular dynamics simulations and analysis

The systems for molecular dynamics (MD) simulations were prepared based on the solution structure 2ROP (the two-domain construct MBD3-MBD4 of human ATP7B) (Banci et al. 2008). The chain A of 2ROP, i.e., MBD4, was extracted as the target. The structure was trimmed based on the sequence in Fig. 1. The residue G386 of the wild type was exchanged in silico to V386 and D386 to generate two MBD4 variants. In total we built 3 systems, namely wild type MBD4, G386V-containing MBD4 (MBD4V) and G386D-containing MBD4 (MBD4D). The hydrogens were added to the proteins and the protonation states were tuned at neutral pH conditions. The prepared structure (wild type system as an example) was solvated in a 66 Å rhombic dodecahedron TIP3P water box (Jorgensen et al. 1983) to ensure 10 Å buffer space between the protein atoms and the boundary of the water box. 0.15 M of NaCl was added to neutralize the system and mimic the physiological conditions. The system was initially minimized for 11,000 steps under a series of position restraints and constraints, then heated to 300 K and equilibrated under NVT condition (constant volume and temperature) for 1 ns using the CHARMM program (version 38a1) (Brooks 2009).

200 ns production MD simulation was carried out at NPT condition (constant pressure and temperature) for each system using the NAMD program (version 2.10) (Phillips et al. 2005). The pressure was controlled by Nosé-Hoover Langevin piston method with 200 ps piston period and 100 ps piston decay time (Feller et al. 1995). The temperature was maintained at 300 K by using Langevin thermostat with 5 ps friction coefficient. Integration time step was set to 2 fs, which is allowed through constraining any bond involving hydrogens by SHAKE algorithm. Non-bonded van der Waals energies were calculated using a switching function with the switching distance from 9 to 11 Å (Loncharich and Brooks 1989), and electrostatic interactions were evaluated using the particle mesh Ewald summation (PME) method (Petersen 1995). The CHARMM36 force field (Best et al. 2012) with the CMAP correction (MacKerell et al. 2004) was used to present the proteins. In addition to the simulations at 300 K, MBD4 and MBD4V were also simulated for 200 ns at 330 K using the same protocol as above. The covariance matrix analysis, principle component analysis (PCA) (Brooks et al. 1995) and geometric measurements on the simulated systems were fulfilled in CHARMM routines. All the figures of the protein structures were prepared by the PyMOL graphic software (PyMOL version 0.99, DeLano Scientific LLC, CA).

## Results

### Secondary structure and thermal stability in vitro

**Fig. 2:**
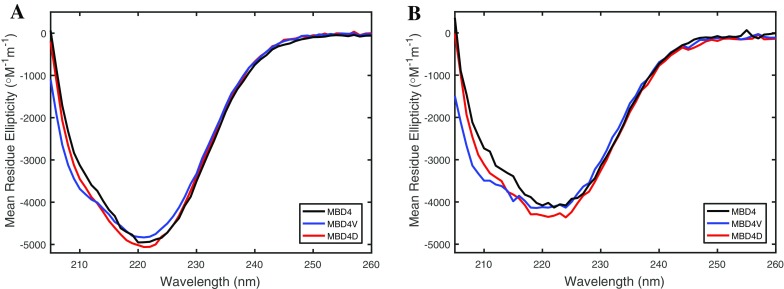
CD spectra of MBD4D (*red*), MBD4V (*blue*), and MBD4 wild-type (*black*): **a** without Cu, and **b** in the presence of Cu in a 1:1 molar ratio of metal to protein

The far-UV CD spectrum at 6 °C of wild-type MBD4 displayed a broad negative band at around 222 nm indicating a mixture of β-sheet and α-helix structures (Fig. 2a), as expected for a ferredoxin-like structure (Banci et al. 2008). The MBD4V and MBD4D mutants also showed characteristic CD spectra of folded structures, very similar to the spectrum of the wild-type protein. MBD4V showed a somewhat more pronounced CD band at 208 nm (that reports on α-helical content) as compared to the other two variants.

**Fig. 3:**
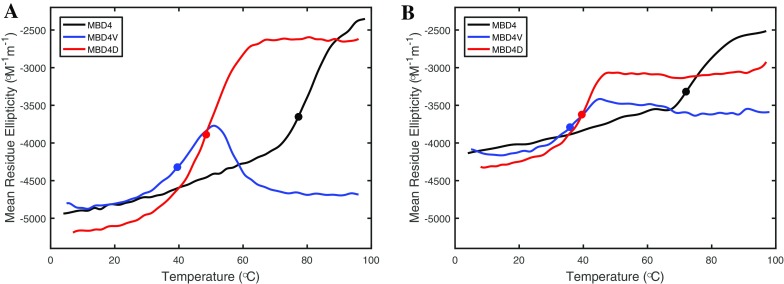
Thermal unfolding profiles of MBD4D (*red*), MBD4V (*blue*), and MBD4 (*black*). without Cu (**a**) and in the presence of Cu in a 1:1 molar ratio (**b**) probed by CD at 222 nm. *Circles* indicate estimated midpoints of the unfolding transitions (the first transition for MBD4V)

**Fig. 4:**
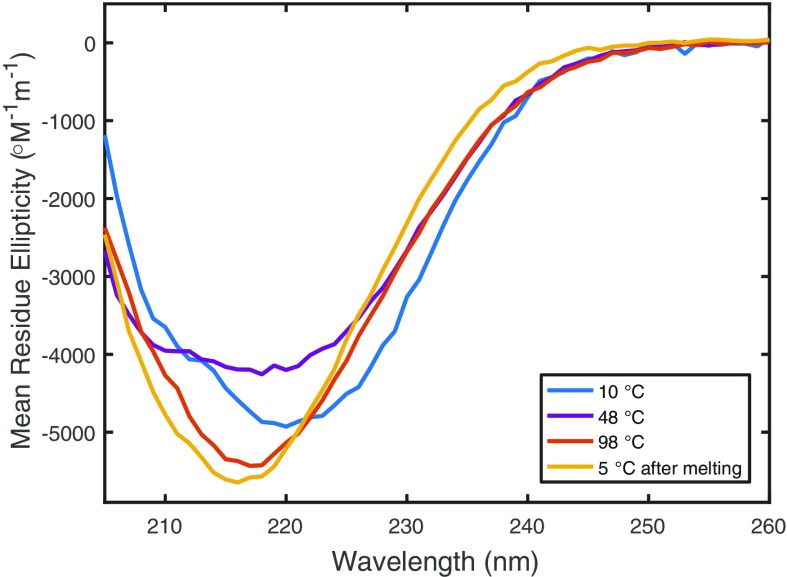
CD spectra of MBD4V at 10 °C (*blue*), 48 °C (*purple*), 98 °C (*red*), and, upon re-cooling of heated sample, at 5 °C (*yellow*)

Thermal unfolding experiments of the apo-forms of the variants probed by CD at 222 nm demonstrated that the wild-type protein unfolded in a partly reversible process, whereas MBD4D unfolded irreversibly (Figs. 3a, S2). The MBD4V variant had a different thermal unfolding profile in which unfolding was followed by refolding into a different structure (Figs. 3a, 4). The first change upon increasing the temperature was unfolding, as reflected in a decreased absolute CD signal at 222 nm. Above 50 °C, unfolding was followed by a significant gain of secondary structure as reflected by an increased absolute CD signal at 222 nm (Fig. 3a). CD spectra at different temperatures during heating of MBD4V showed that at 48 °C, the spectrum was characteristic of a partially-unfolded protein, whereas at higher temperatures, the spectrum was indicative of β-sheet structure (Fig. 4). We note that heating-induced protein misfolding into structures with large secondary structure content has previously been documented for other proteins (Pozdnyakova and Wittung-Stafshede 2010; Sedlak et al. 2008).

Despite the anomalous misfolding of the MBD4V variant, it is clear that both mutants had dramatically lower thermal stability (defined as the temperature at which the folded structure is lost) than the wild-type MBD4 (Fig. 3a). We estimated the thermal midpoints (T_m_) of the mutant unfolding transitions to be in the range of 40–50 °C (i.e., where 50 % of the protein sample is unfolded), whereas wild-type MBD4 had a thermal midpoint above 75 °C. Thus, even though the mutations are located in a loop at the surface of the folded protein (Fig. 1), they have pronounced effects on the overall thermal stability of the MBD4 structural fold.

Both mutants, like the wild-type domain (Niemiec et al. 2012, 2015), coordinated Cu stoichiometrically when mixed in micromolar concentrations in the presence of 5-fold excess DTT. This is reasonable since the mutation site is opposite to the Cu-binding site. The spectra of the holo-forms are similar to those of the apo-forms with only small changes in the CD intensity (Fig. 2b). Thermal denaturation experiments demonstrated that, when Cu was loaded onto the protein, the T_m_ of the unfolding reactions decreased for all the variants by 5–10 °C at our conditions when compared to the apo-forms (Fig. 3b). Thermal unfolding reactions of Cu-loaded proteins were, like for the apo-forms, partially reversible for wild-type MBD4 but irreversible for the mutants, based on CD data collected upon cooling (Fig. S3). The increase of the absolute CD value at 222 nm seen at higher temperatures for apo-MBD4V was not observed for the holo-form of MBD4V. However, the spectrum of holo-MBD4V after re-cooling was, as in the case of apo-MBD4V, characteristic of β-sheet structure (Fig. S3).

### Structural dynamics and protein motion in silico

To probe the influence of the mutations at atomistic level, we performed 200 ns MD simulations for each of MBD4, MBD4V and MBD4D in apo-forms. Because all-atom simulations of complete protein folding/unfolding processes at room temperature are still computationally challenging (Scheraga et al. 2007) we here focused on variations in local structural dynamics and motions of the folded protein domain. Unless specified, analyses are based on simulations at 300 K.

**Fig. 5:**
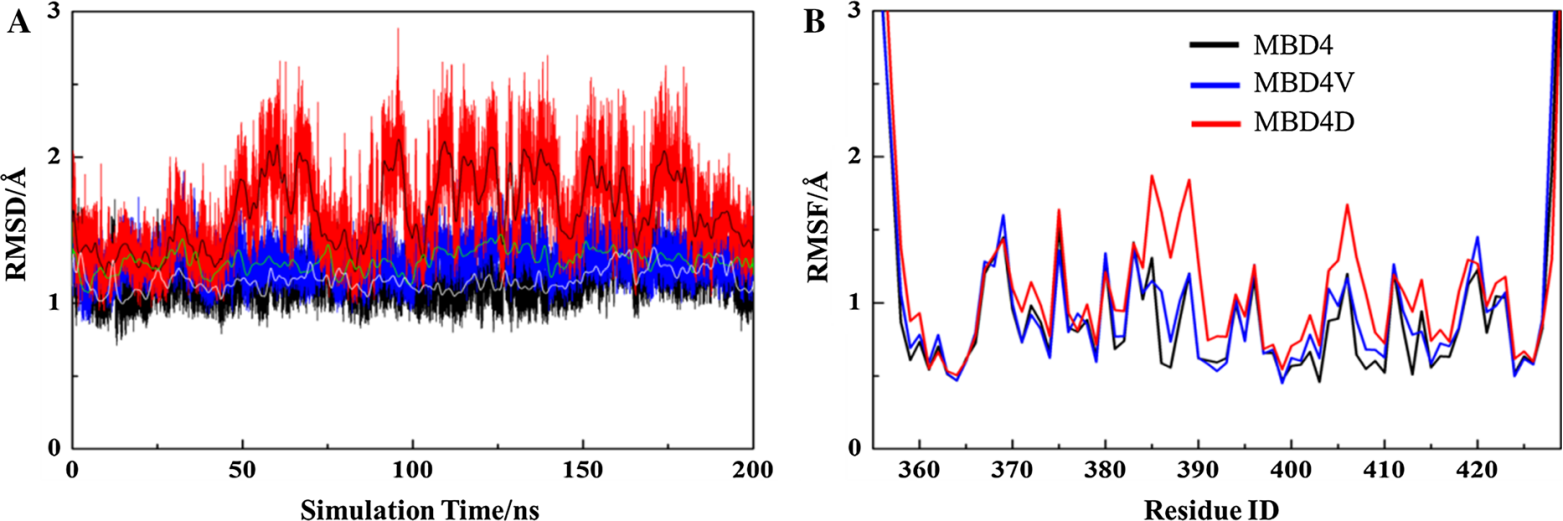
Structural deviation and fluctuation of MDB4 proteins. **a** Backbone RMSD profiles of MBD4, MBD4V and MBD4D (*black*, *blue* and *red*, respectively). The smoothed curves are shown together with their corresponding RMSD curves. **b** Heavy atom RMSF profiles with the same color scheme as in (**a**)

The root-mean-square-deviation (RMSD) profiles showed that MBD4V and MBD4D, especially MBD4D, experienced more pronounced structural deviations than MBD4 (Fig. 5a). The RMSD of MBD4D periodically deviated 2.5 Å away from its minimized solution structure after 50 ns, suggesting a substantial perturbation induced by the introduced Asp at position 386. The root-mean-square fluctuation (RMSF) profiles located the perturbed regions, which corresponded to the residues around the mutated 386 position (the loop connecting α1 and β2) and part of β2 ranging from residue 403 to 410 (Fig. 5b). The secondary structure analysis also showed that MBD4D had lost β structure (Fig. S4a–c) which was attributed to the distortion of β2 (Fig. S4d). Taken together, there is augmented structural dynamics in silico upon mutation at position 386, with an Asp substitution causing more distortion than a Val substitution.

**Fig. 6:**
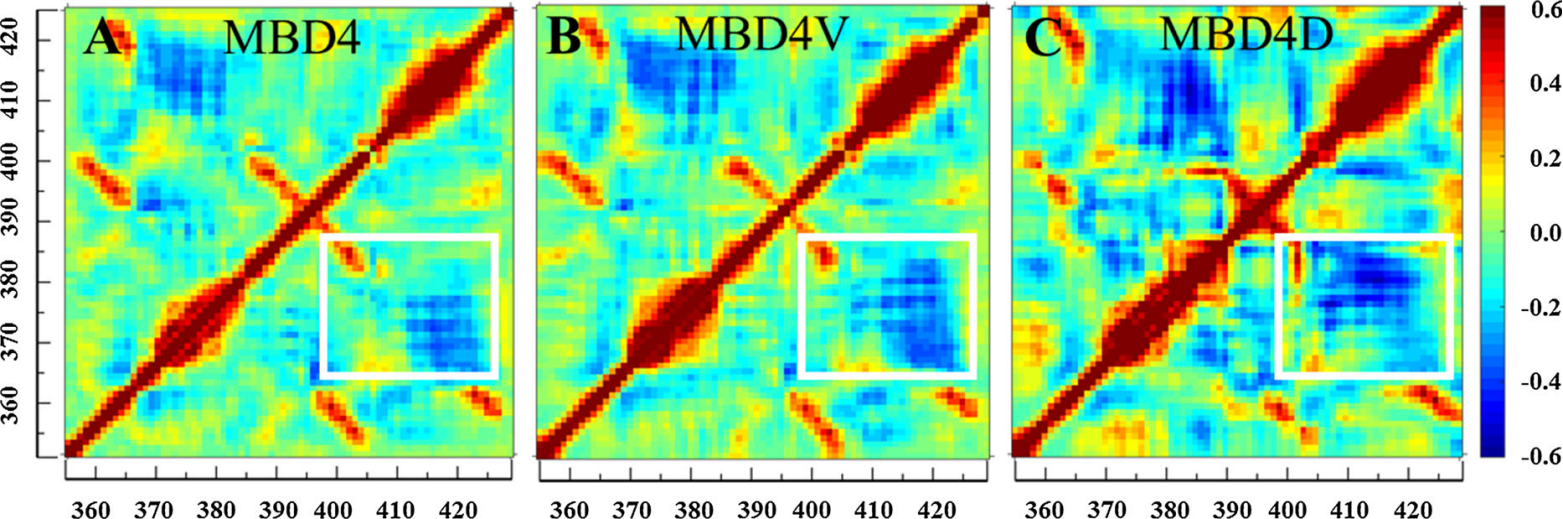
Covariance matrices of MBD4 (**a**), MBD4V (**b**) and MBD4D (**c**) with the X and Y axes reporting on residue ID. The color scale changes from *blue* (−0.6 and smaller correlation value) for anti-correlation to *green* (zero) for non-correlation and to *red* (0.6 and greater correlation value) for correlation between a pair of residues

To explore how the mutations influence protein motions, we employed covariance matrix analysis and PCA. Two major differences in the correlated motions between the wild-type MBD4 and two variants were found. First, pair-wised correlations along the matrix diagonal were strengthened in MBD4V and MBD4D as compared with in MBD4. Second, the mutations produced enhanced anti-correlations throughout the whole MBD4V and MBD4D structures (Fig. 6a–c). The structural regions whose anti-correlations were mostly enhanced (boxed in Fig. 6) in MBD4V and MBD4D corresponded roughly to helices α1 and α2. The enhanced motions included shearing and breathing motions of the two helices and were structurally captured by PCA (arrows, Fig. S5). Pronounced motions may make structural units, such as α1 and α2, more prone to large-scale conformational changes.

To trace the origin of MBD4V’s misfolding at high temperatures found in vitro, we also performed MD simulations on MBD4 and MBD4V at 330 K. During these simulations, MBD4V underwent larger fluctuations than wild type MBD4 (Fig. S5). After 120 ns, MBD4V experienced a significant deviation relative to its original structure (Fig. S6a), suggesting large-scale conformational changes taking place. The RMSF profile showed that structural changes occurred in the end of α1 and in the loop between α1 and β1 (Fig. S6b). Investigation of the trajectory showed that MBD4V had lost part of α1, resulting in an extended loop between α1 and β1 (Fig. S6c, d). This elongated loop may facilitate the transition to the β-sheet-rich structure (as found experimentally).

## Discussion

The mutations G85V and G591D result in Wilson disease in multiple affected family members (Loudianos et al. 1998). Because this position is highly conserved among the human MBDs, this Gly residue, despite its surface-located position, must be important for the overall ATP7B function in vivo. The question is why and how this is explained. Our experiments demonstrate that mutation of this Gly to Val or Asp in MBD4 creates pronounced domain thermal destabilization in vitro and enhanced domain structural dynamics in silico. All MBD4 variants are stable (i.e., folded and capable of binding Cu) at temperatures below 10 °C but the two mutants unfold at considerably lower temperatures as compared to wild type MBD4. The lowered thermal stability of the mutants (regardless of Cu loading or not) and the irreversibility of the reactions suggest that, at physiological temperatures, such mutated domains will be largely unfolded. The observation that MBD4V misfolded into a new β-sheet-rich species at high temperatures indicates that the MBD structural unit is prone to alternate structures and that a small increase in hydrophobicity, such as upon introducing a Val, may act as a trigger.

Although several Wilson disease mutations result in defects in ATP7B trafficking, ATP7B proteins with a G85V or G591D mutation were reported to have normal cellular localization (although this has been questioned (de Bie et al. 2007)), but they appear to be expressed at lower levels (de Bie et al. 2007; van den Berghe et al. 2009) and have impaired interactions with Atox1 in model systems (Hamza et al. 1999). If conformational changes are strongly coupled among the six MBDs, or/and between MBDs and other domains in ATP7B, partial/full unfolding and/or increased structural dynamics of an individual MBD may cause overall protein dysfunction, although occurring distant from the functional sites. Molecular studies of full-length proteins harboring these mutations are required to reveal what functional step(s) (e.g., Cu uptake from Atox1, ATP binding and/or hydrolysis, Cu movement along the MBDs or through the membrane channel, vesicle trafficking) are primarily affected by MBD1 and MBD6 instability. It is also important to assess if unfolding of one mutated domain within the full-length protein remains a local phenomenon, or if this triggers unfolding of other domains as well, resulting in wide-spread perturbation of the protein.

Interestingly, both G85V and G591D mutated ATP7B variants were found to have increased COMMD1 interactions (de Bie et al. 2007). It has been proposed that COMMD1 is a negative regulator of protein stability and its binding to the two ATP7B variants may thus be part of the cellular quality control system (de Bie et al. 2007). In vivo, COMMD1 interactions may block ATP7B Cu-transport activity and/or direct ATP7B for degradation. Thus one scenario is that instability/unfolding of MBD1 or MBD6 triggers COMMD1 binding that, in turn, sterically block Cu transport activity of the protein.

In summary, introducing a larger amino acid (Asp or Val instead of Gly) at position 386 in MBD4 (as found in MBD1 and MBD6 in Wilson disease patients) results in enhanced domain structural dynamics in silico and decreased resistance towards thermal perturbations in vitro. Our findings suggest that these two mutations cause Wilson disease in patients because of MBD structural instability that indirectly or directly affects domain–domain interactions in ATP7B which, in turn, impair the overall Cu-transport function.

## Electronic supplementary material

Below is the link to the electronic supplementary material.

Supplementary material 1 (DOCX 1415 kb)
