# Supplementary material for: Disease-causing point-mutations in metal-binding domains of Wilson disease protein decrease stability and increase structural dynamics
Source: Biometals. 2016 Oct 15;30(1):27–35. doi: 10.1007/s10534-016-9976-7 (PMC5285417; doi:10.1007/s10534-016-9976-7)
Supplement: Supplementary file 1 — Supplementary material 1 (DOCX 1415 kb) [file 10534_2016_9976_MOESM1_ESM.docx]

**Supplementary Information**

**Disease-causing point-mutations in metal-binding domains of Wilson disease protein decrease stability and increase structural dynamics**

*Ranjeet Kumar, Candan Ariöz, Yaozong Li, Niklas Bosaeus,*

*Sandra Rocha, and Pernilla Wittung-Stafshede*

**Content:**

**Figures S1-S6**

**Supplementary Figures**


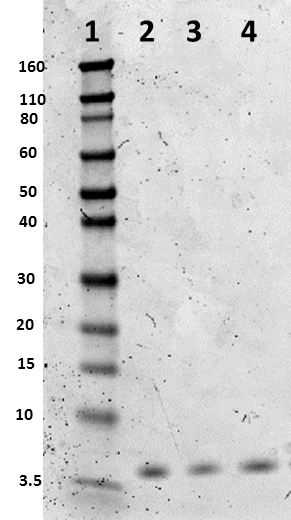


**Figure S1.** Purified proteins shown on a 12% SDS-PAGE gel. Lanes 1 to 4 represent molecular weight markers (sizes given in kDa), MBD4, MBD4D and MBD4V, respectively.

| A  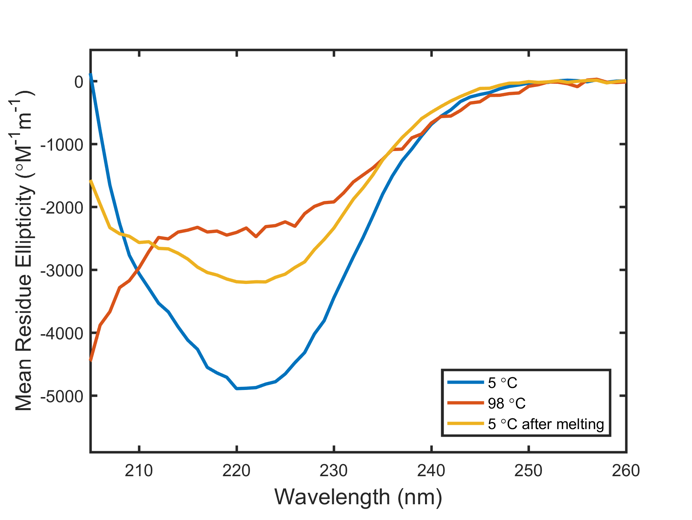 | B  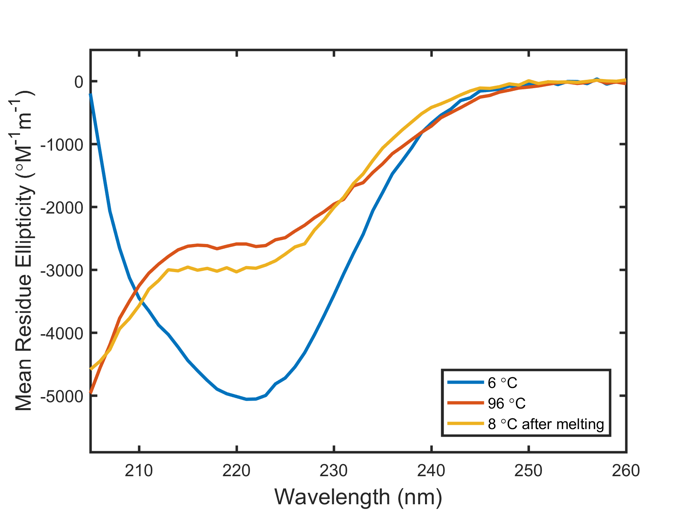 |
| --- | --- |
|  |  |

**Figure S2**. CD spectra of apo-form proteins before heating (blue), at the highest temperature (red), and after re-cooling (yellow) for MBD4 (**A**) and MBD4D (**B**).

**
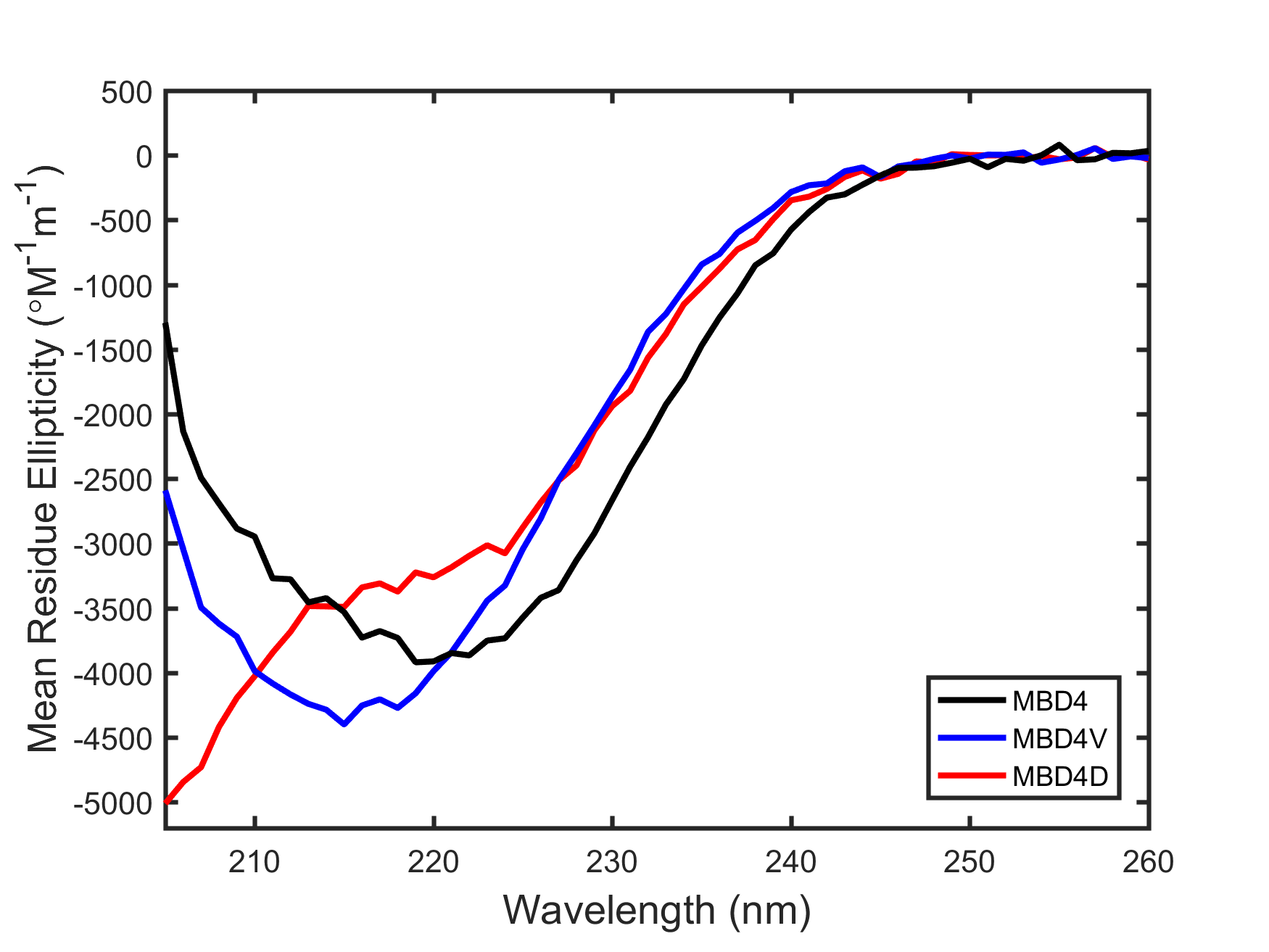
**

**Figure S3.** CD spectra of Cu-loaded forms of the proteins collected at 5 °C after completion of the thermal (heating-cooling) cycle.


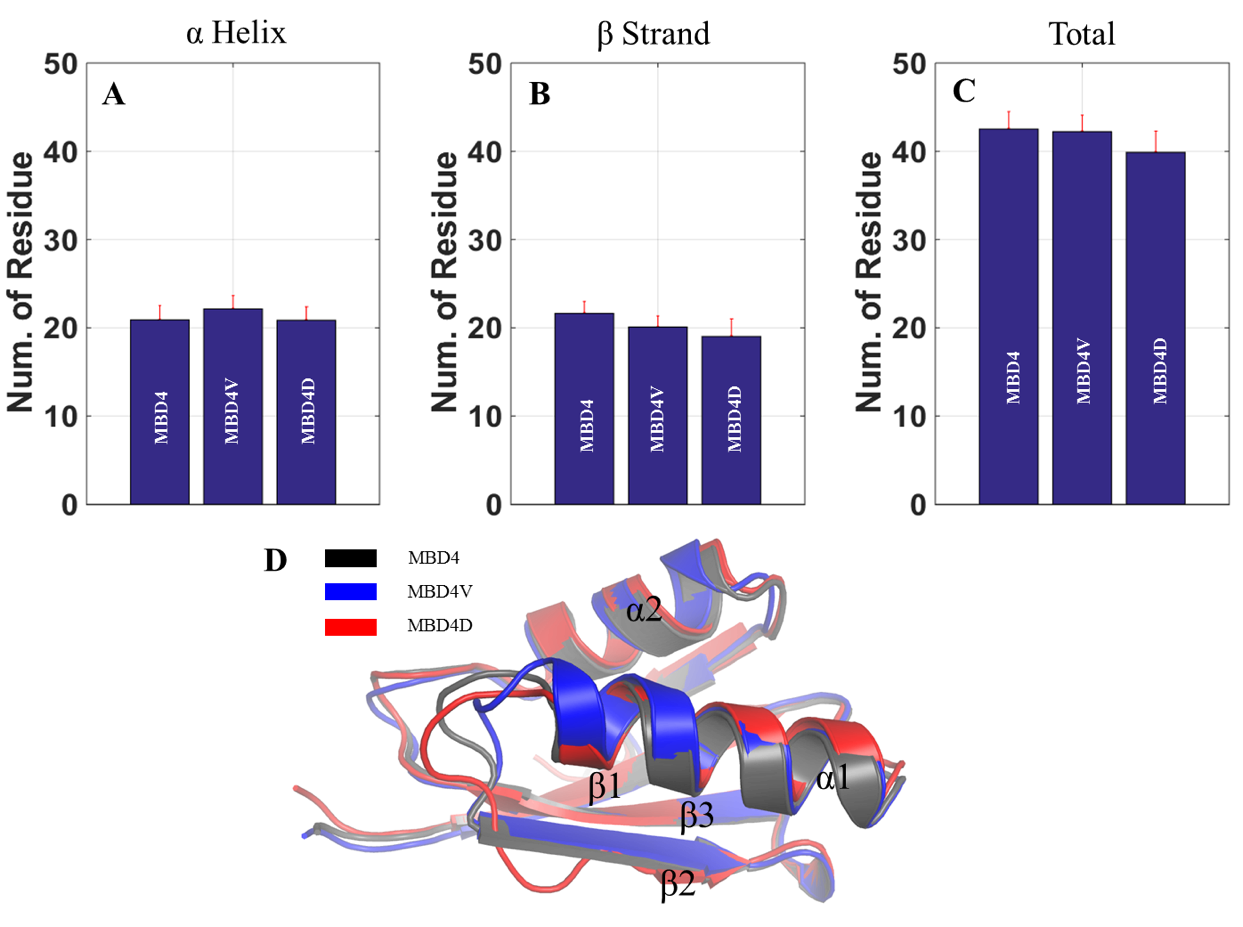


**Figure S4**. Secondary structure content from in silico simulations (A, α-helix; B, β-strand and, C, total) shown as average number of residues that constitutes a specific secondary structure (error bar indicates fluctuation during the simulation). D. Structural alignment of the average structures that were generated based on 100,000 snapshots from the 200 ns simulation. MBD4 (black), MBD4V (blue) and MBD4D (red).


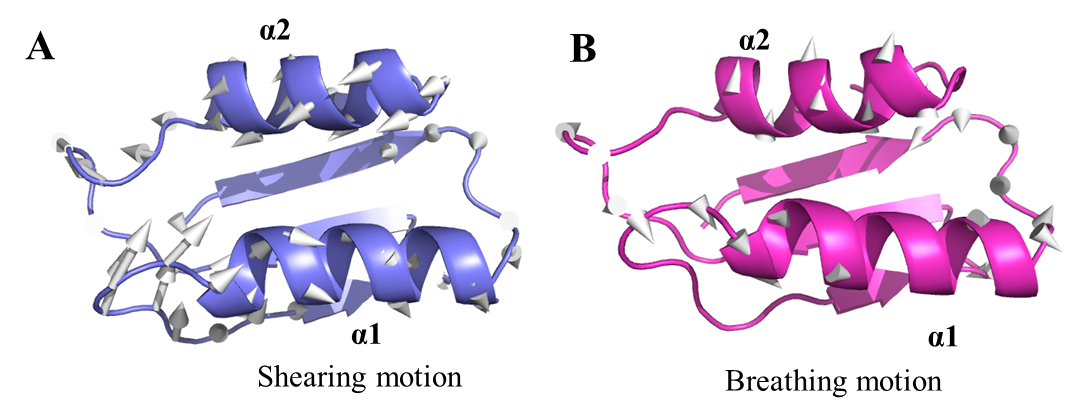


**Figure S5.** Protein motions from PCA reveal shearing (A) and breathing (B) motions, which mainly involve helices α1 and α2. Relative movements between these helices (gray arrows).


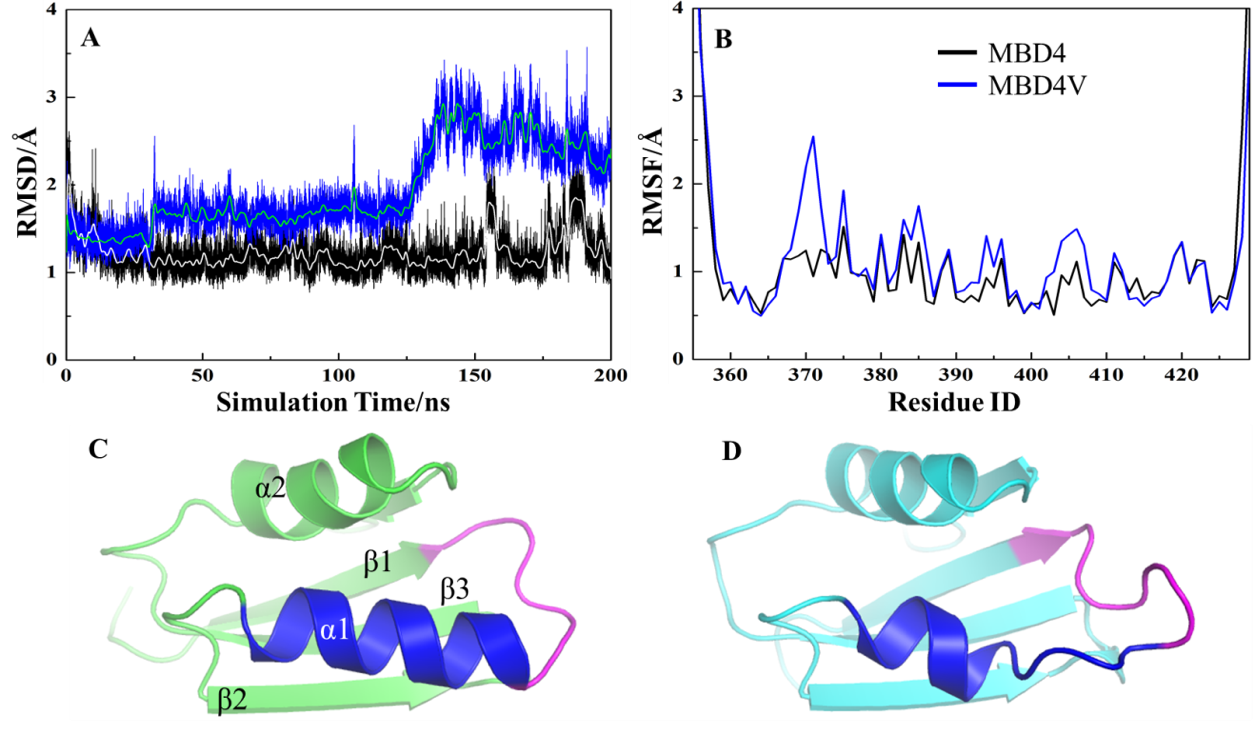


**Figure S6.** MD simulations of MBD4 and MBD4V at higher temperature. A. Backbone RMSD profiles of MBD4 (black) and MBD4V (blue) are shown as smoothed curves and actual raw data. B. Heavy atom RMSF profiles of MBD4 (black) and MBD4V (blue), with presentation of the first (C) and last (D) frames of the MBD4V trajectory.
